# Neuropsychological Changes in Complex Regional Pain Syndrome (CRPS)

**DOI:** 10.1155/2020/4561831

**Published:** 2020-01-14

**Authors:** Monika Halicka, Axel D. Vittersø, Michael J. Proulx, Janet H. Bultitude

**Affiliations:** ^1^Centre for Pain Research, University of Bath, Claverton Down Road, Bath BA2 7AY, UK; ^2^Department of Psychology, University of Bath, Claverton Down Road, Bath BA2 7AY, UK; ^3^Department of Sport & Health Sciences, University of Exeter, Prince of Wales Road, Exeter EX4 4SB, UK; ^4^Centre for Real & Virtual Environments Augmentation Labs, Department of Computer Science, University of Bath, Claverton Down Road, Bath BA2 7AY, UK

## Abstract

Complex Regional Pain Syndrome (CRPS) is a poorly understood chronic pain condition of multifactorial origin. CRPS involves sensory, motor, and autonomic symptoms primarily affecting one extremity. Patients can also present with neuropsychological changes such as reduced attention to the CRPS-affected extremity, reminiscent of hemispatial neglect, yet in the absence of any brain lesions. However, this “neglect-like” framework is not sufficient to characterise the range of higher cognitive functions that can be altered in CRPS. This comprehensive literature review synthesises evidence of neuropsychological changes in CRPS in the context of potential central mechanisms of the disorder. The affected neuropsychological functions constitute three distinct but not independent groups: distorted body representation, deficits in lateralised spatial cognition, and impairment of non-spatially-lateralised higher cognitive functions. We suggest that many of these symptoms appear to be consistent with a broader disruption to parietal function beyond merely what could be considered “neglect-like.” Moreover, the extent of neuropsychological symptoms might be related to the clinical signs of CRPS, and rehabilitation methods that target the neuropsychological changes can improve clinical outcomes in CRPS and other chronic pain conditions. Based on the limitations and gaps in the reviewed literature, we provide several suggestions to improve further research on neuropsychological changes in chronic pain.

## 1. Introduction

Complex Regional Pain Syndrome (CRPS) is a chronic pain condition of poorly understood origin that predominately affects distal parts of one extremity, although in some cases it can spread to other limbs over time [1]. It is characterised by a combination of sensory, motor, and autonomic abnormalities. There is growing body of evidence suggesting that despite the absence of any brain lesions, people with CRPS can present with neuropsychological symptoms. Previous reviews have attempted to address the topic of “neglect-like” symptoms (e.g., spatial attention bias away from the CRPS-affected side [2–4]). Going beyond the analogy to hemispatial neglect and integrating the current knowledge about the full breadth of cognitive changes found in CRPS is important for elucidating the cortical and cognitive mechanisms that could be involved in the development, maintenance, and treatment of its clinical symptoms. This might have implications for other chronic pain conditions that share similar neuropsychological components. Therefore, this article provides a comprehensive, critical review of the evidence for altered neuropsychological functions in CRPS.

We conducted a literature search using the PubMed database for articles including keywords regarding Complex Regional Pain Syndrome published in English between 1995 and 2019. To identify relevant articles, we screened the titles and abstracts for keywords regarding cognitive function. We also manually searched and cross-referenced the reference lists of relevant articles to identify additional studies that were not detected through the initial literature search. Because the clinical presentation and recovery rates of paediatric CRPS differ from CRPS in the adult population [5–7], we limited the scope of this review to adults. However, it is noteworthy that we did not identify any studies investigating neuropsychological changes in children with CRPS in the literature search.

Integrating the existing evidence for neuropsychological changes in CRPS, in the current review, we do the following:

- Summarise the clinical presentation of CRPS and proposed pathophysiological mechanisms, including peripheral and central processes, with the aim to situate the neuropsychological symptoms in the clinical picture of the syndrome
- Review the evidence of neuropsychological changes in CRPS, distinguishing three major categories: body representation disturbances, lateralised spatial cognition deficits, and non-spatially-lateralised higher cognitive deficits. Where applicable, we relate these symptoms to evidence of similar cognitive deficits in people who suffered brain lesions or other chronic pain conditions
- Discuss the specificity of neuropsychological symptoms to CRPS and their clinical relevance with regard to the development, maintenance, and treatment of CRPS

We conclude that the currently used “neglect-like” framework is insufficient for characterising the variety of neuropsychological changes shown by people with CRPS and advocate the role of parietal cortical networks in the emergence of these symptoms.

## 2. Clinical Features and Pathophysiology of CRPS

CRPS most commonly develops following a fracture, sprain, or surgery, although there are known instances of spontaneous onset [8–10]. Persistent, continuing pain disproportionate to any preceding injury is the primary complaint, but CRPS also affects a range of other physical and cognitive functions. In the following sections, we summarise the clinical manifestations of CRPS and their proposed pathophysiological mechanisms, to provide context for understanding the changes in higher cognitive functions in these patients.

### 2.1. Sensory, Autonomic, and Motor Symptoms

The diagnosis of CRPS requires both self-reported symptoms and signs that are evident during clinical examination [11] (see diagnostic criteria in Table 1). Sensory changes include perceiving nonnoxious stimulation as painful (allodynia) and/or experiencing severe or prolonged pain in response to mildly noxious stimulation (hyperalgesia). Autonomic dysfunction can manifest as temperature, skin colour, and sweating asymmetry between the affected and unaffected limbs, oedema, and changes in skin appearance and hair and nail growth on the affected extremity. Motor abnormalities include tremor, decreased range of movement, muscle weakness, and/or having the affected limb set in a sustained, fixed posture (dystonia). The breadth of clinical manifestations and their possible combinations means that CRPS is a multifaceted and heterogeneous disease.

### 2.2. Peripheral and Central Mechanisms of CRPS

The pathophysiology of CRPS is not well understood, and evidence points towards a multifactorial origin of this disorder. The most strongly implicated mechanisms can be classified into peripheral and central processes (for reviews, see [13–16]). In brief, an aberrant inflammatory response to tissue trauma can lead to sensitisation of peripheral and spinal nociceptive fibres, neuroinflammation, and dysfunction of peripheral blood circulation [17–20]. Peripheral mechanisms cannot fully account for the fact that CRPS symptoms persist long after the inflammatory response should have resolved. However, patients also show maladaptive plastic changes in the central nervous system [16, 21, 22]. Changes on the spinal and supraspinal level directly linked to clinical signs of CRPS involve central sensitisation, whereby spinal nociceptive neurons become hyperresponsive to peripheral input and increase nociceptive signalling to the cortex even in the absence of such input [23–26]. A shift from inhibition towards facilitation of nociceptive input was also found in the endogenous pain modulation system in CRPS [27]. Peripheral and central mechanisms are not contradictory, and they can interact to produce clinical signs of CRPS. Central changes also occur at a higher, cortical level [16, 28]. The evidence regarding structural reorganization is scarce [29, 30], but extensive evidence of functional cortical reorganization of sensory and motor representations of the limbs in CRPS has been reviewed elsewhere [31, 32]. This review concerns behavioural and clinical evidence for altered higher cognitive functions (i.e., neuropsychological symptoms), which thus far have not been comprehensively summarised.

## 3. Altered Neuropsychological Functions in CRPS

In the following section, we review higher cognitive processes that are affected in CRPS and that suggest cortical reorganization. The known physiological underpinnings of CRPS alone cannot account for some cognitive phenomena observed in this condition, though neuropsychology provides a useful framework for explaining them. The neuropsychological changes include body representation distortions (Section 3.1), lateralised spatial cognition deficits (Section 3.2), and other neuropsychological symptoms that implicate disruption of broad cortical networks, especially parietal functioning (Section 3.3). We summarise and discuss the study details and behavioural findings from research investigating these neuropsychological changes in CRPS (see also Table 2).

### 3.1. Body Representation

Altered body representation is among the earliest and best characterised neuropsychological changes in CRPS. Cognitive representations of one's body are derived from proprioceptive, vestibular, somatosensory, and visual information that interact with the motor system to guide actions [79]. This dynamic online representation of body posture is often called “body schema” [80]. However, in this review, we use a broader term “body representation” that also incorporates the structural definition of the body (i.e., perception of its size, shape, and boundaries) as well as the body image (defined as the semantic representation of the names and function of distinct body parts) [80]. Distortions of body representation manifest in CRPS as self-reported disturbed perceptions, ownership of and feelings towards the affected limb; difficulties with mentally rotating and recognising the laterality of pictures of the limbs; and erroneous estimation of the size, position, and movement of the limbs from single sensory modalities (while multisensory integration appears intact). Below we discuss evidence for each of these manifestations in turn.

#### 3.1.1. Self-Reported Body Perception Disturbances

Initial clinical reports [33] and questionnaire studies [36, 37] showed that up to 60% of patients reported loss of ownership, recognition, or awareness of their CRPS-affected limb. This research is aimed at measuring the so-called “neglect-like” symptoms in CRPS. Neglect is an attention deficit affecting the hemispace contralateral to a brain lesion [81], discussed in more detail in Section 3.2. Early research in CRPS considered reports of the affected limb not being part of the patient's body and feeling dead as “cognitive neglect” symptoms [35, 36], yet we would argue that they are better characterised as a disturbance of the mental representation of the body. Specifically, these symptoms closely resemble asomatognosia (lost sense of ownership of one's limb), which can follow temporoparietal lesions. Asomatognosia often cooccurs with hemispatial neglect, yet it is not a diagnostic feature of the neglect syndrome [82, 83]. Interviews of people with CRPS about their perceptions of their body [34] revealed a range of disturbances consistent with distorted body representation (see also [52]). These included perceptions of the affected limb as being larger or smaller, misshapen, or heavier relative to its true size, shape, and weight; negative feelings towards the affected limb such as disgust or hatred (reminiscent of misoplegia [84]); the desire to amputate it; a mismatch between sensation of the affected limb and its appearance; lacking parts of the limb from their mental representation; and poor awareness of the affected limb's position. Although more prevalent in chronic CRPS [37], such experiences can manifest within days of disease onset [34]. The severity of self-reported body perception disturbance correlated with impaired tactile acuity [47], which was linked to reorganization of the primary and secondary cortical maps of the CRPS-affected limb [85, 86]. This suggests that subjective body representation distortion could be accompanied by changes in the brain pertaining to the central mechanisms of CRPS.

#### 3.1.2. Limb Laterality Recognition

Several studies have used variations of the limb laterality recognition task, also sometimes referred to as mental hand/foot rotation, to measure body schema in CRPS (e.g., [45, 57–59, 61–63]). In a typical procedure, the task requires speeded identification of left or right limbs from pictures of hands or feet in different postures and/or at different angles of rotation from the upright (canonical) position. In pain-free controls, response times increase with the angle of rotation (i.e., they get longer consistent with the spatial disparity between the pictures of limbs and the canonical posture and also according to the biomechanical constraints that make some hand rotations physically easier than others [87]). Therefore, it is thought that the limb laterality recognition task involves mentally rotating the pictured limb to match it to the current position of one's own limb (or vice versa) in a manner that complies with biomechanical constraints [59, 88, 89]. This is thought to require the participants to use the cognitive representations of the limb that corresponds to the one depicted in the picture [90, 91]. Consistent with the involvement of motor imagery [87], neuroimaging studies show increased activation of premotor and parietal regions during hand laterality recognition [92, 93].

People with CRPS were less accurate and slower in determining the laterality of images corresponding to their painful limb than of images corresponding to their unaffected limb [56–60], indicative of the cognitive representation of the affected limb being distorted. Moreover, Reid et al. [58] found that in addition to taking longer to recognise pictures of limbs corresponding to their affected side of the body, people with CRPS took longer to recognise pictures of limbs presented in their affected side of space. The latter effect occurred for both the images of hands and feet regardless of whether participants had CRPS in upper or lower limbs; however, it was specific to images of body parts and not to other stimuli (e.g., letters). Although there appears to be strong evidence for lateralised body representation distortions in CRPS, some authors have reported equally slowed limb laterality judgements for pictures representing both the affected and unaffected limbs, compared to healthy controls [42, 45, 61, 62]. This could be due to methodological differences, or it could indicate more generalised changes in body representation or reduced psychomotor speed due to the effects of pain medication [94] or chronic pain in general (rather than CRPS specifically) [95]. This would be consistent with the finding of comparable slowing in laterality recognition of both limbs in phantom limb pain and CRPS [45, 62]. Finally, there are also contradictory findings suggesting that both people with CRPS and healthy controls are faster in recognising the images of limbs corresponding to their dominant hand, regardless of which side of the body is affected [40] or do not differ in limb laterality recognition [63].

#### 3.1.3. Estimation of Limb Size, Position, and Movement from Unisensory Cues

Distorted perceptions of the body are evident in several modalities, including its visual and proprioceptive representations. Patients with CRPS were presented with compressed and expanded schematic drawings of hands [50] and real pictures of their own hands manipulated in the same manner [49]. When asked to indicate the pictures that most accurately represented the size of their affected hands, they tended to choose enlarged images, overestimating the size of their painful extremities.

Distorted estimates of limb position and limb movement have also been reported for people with CRPS. “Manual” or “proprioceptive straight-ahead” [96] requires participants to point straight ahead of their perceived body midline, without vision of the limb or external space (e.g., with the eyes closed), and thus relies on integrating proprioceptive information about position of an arm with perceived body midline. A shift of manual straight-ahead towards the affected side of space relative to objective midline has been found in a case of CRPS [53, 54] when the patient used the affected hand and also when she used the unaffected one. Nevertheless, two group studies found no significant deviations from the true body midline nor from the subjective midline of healthy and pain controls, on the same manual task performed with either or both arms [38, 55]. Manual straight-ahead estimations of individuals with CRPS were not more variable than among the controls [38]. However, people with CRPS presented with impaired limb position sense in two studies that used matching tasks. In Lewis et al.'s [52] study, participants were required to match the position of their affected and unaffected arm to specified targets that were *external* to their body (i.e., point their arms as though they were the hour hand on a clock showing a particular time). In Brun et al.'s [44] study, they were required to match the position of the affected or unaffected arm to the mirror-reverse position of their other arm, which had been passively moved by a robot. In both of these studies, people with CRPS made more errors and were less precise than healthy controls when positioning both arms when they did not have vision of their limbs. This suggests that proprioceptive deficits are bilateral and thus cannot be attributed solely to sensory deficits in the CRPS-affected limb.

In a third task, people with CRPS also presented with reduced accuracy and precision in the sense of limb *movement*. Participants observed movement of a virtual limb anchored to the movement of their unseen affected limb and judged whether it was smaller or greater than their actual movement. People with CRPS both under- and overestimated the extent of their movements relative to healthy controls [44]. Both this impaired sense of movement of the affected limb and the previous findings of more variable positioning performance for the affected and unaffected limbs provide evidence of impaired proprioception, since participants could not see their limbs and thus were forced to rely on proprioception for these tasks [44, 52–54]. However, these deficits are not consistently found [38, 55].

#### 3.1.4. Multisensory Contributions to Body Representation in CRPS

Research also investigated how information from multiple sensory modalities is combined to contribute to body representation in CRPS. An additional observation from the study by Lewis et al. [52] is that when people with CRPS kept their eyes open while they placed their affected arm at particular clock face locations, their limb position deficits were smaller than when they performed the task with their eyes closed. Positioning of the unaffected arm did not significantly improve with vision. This demonstrates that people with CRPS rely on visual cues in addition to proprioceptive ones when estimating the position of the affected limb. Furthermore, Tajadura-Jiménez et al. [48] found that the self-reported inability to visualize the affected limb or overestimation of its size could be altered by auditory feedback during movement. In this study, people with upper or lower limb CRPS heard manipulated sounds linked to their footsteps, with higher frequencies inducing an impression of lighter body weight and smaller body dimensions and lower frequencies inducing an impression of heavier weight and larger body dimensions. Similar to the performance of healthy participants in another study [97], the gait of people with CRPS was altered in that the time of foot contact with the floor increased with lower frequency sounds, consistent with having heavier body. For some participants, the sound feedback also helped to restore the representations of previously missing parts of their body. The studies of Lewis et al. [52] and Tajadura-Jiménez et al. [48] suggest that people with CRPS can integrate visual and auditory feedback with proprioceptive information from their body into the body representation.

However, the process of updating body representation might differ for the affected and the unaffected side. In a recent study, Vittersø et al. [51] demonstrated altered updating of body representation following tool use for people with CRPS compared to controls. Participants estimated the felt distance between two points touching the arm before and after tool use. Tool use typically leads to a shortening of the felt distance between the two points, which is interpreted as a perceived lengthening of the arms as the body representation is updated to incorporate the tools. Relative to pain-free controls, people with upper limb CRPS had a more pronounced updating of body representation for their unaffected arm following tool use (i.e., a larger perceived lengthening than the controls) and showed the opposite pattern for their affected arm (i.e., a perceived shortening). These findings suggest that the representation of the body is more malleable for people with CRPS and that multisensory information can have different effects for the affected and unaffected limbs.

Susceptibility to body-related multisensory illusions can provide insights into which mechanisms governing body representation might be disrupted or preserved in CRPS. The rubber hand illusion is a phenomenon thought to indicate that body ownership arises from integrating congruent visual and tactile input with the existing mental representation of one's body [98]. Thus, preserved multisensory integration should be necessary for illusory ownership of the rubber hand to occur. During the rubber hand illusion, a participant views a real-size rubber arm placed where their real arm would normally reside, while their real arm is placed out of sight nearby and in an analogous orientation [98]. The experimenter applies tactile stimulation (e.g., strokes from paintbrushes) to the rubber and real hand synchronously. There are three classic measures of successful induction of the rubber hand illusion: subjective ownership of the rubber hand; skin conductance responses to viewing the rubber hand being harmed; and a proprioceptive drift of the felt position of the real hand towards the position of the rubber hand. In a study that used the first two of these measures, Reinersmann et al. [41] demonstrated that people with CRPS were able to experience this illusion normally both when the affected and unaffected limbs were stimulated. Specifically, their subjective ownership of the rubber hand and skin conductance responses were not significantly different from those of people with other types of upper limb pain and pain-free controls [41]. We can draw two main conclusions from these findings: people with CRPS can experience an illusory ownership of an artificial limb and they have intact multisensory integration.

Successful induction of rubber hand illusion [41] showed that people with CRPS have the normal ability to perceive an illusory ownership of an artificial body part, despite their decreased sense of ownership of their own affected limb reported in other studies [36, 37]. In Reinersmann et al.'s [41] study, the strength of the illusion was not significantly related to the subjective distortion of body representation as measured by the “neglect-like” symptoms questionnaire [35], which also includes questions about perceived ownership of the painful limb (although see their analysis of a subgroup of right-CRPS participants who reported more distorted perception of their affected limb and weaker ownership of a rubber hand than left-CRPS participants [41]). This is consistent with the findings that the perceived ownership of a rubber hand does not necessitate a disownership of one's real hand [99]. Because these two phenomena appear to be independent, people with CRPS could have normal susceptibility to rubber hand illusion [41] and still experience a decreased sense of ownership of their own affected limb, as reported in other studies [36, 37].

The second conclusion that can be drawn from Reinersmann et al.'s [41] study is that people with CRPS have an intact ability to integrate visual and tactile information (because they have normal susceptibility to the rubber hand illusion). Consistent with this finding, the aforementioned tool use study by Vittersø et al. [51], showing more pronounced updating of bodily representations, also demonstrated intact visuotactile integration in participants with CRPS. These two studies suggest that the multisensory mechanisms that contribute to body representation are intact. Thus, a deficit in multisensory integration per se does not seem to be a plausible explanation for distorted body representation in CRPS. Alternatively, a specific impairment in integration of *proprioceptive* information with other sensory inputs could drive these distortions. People can experience subjective ownership of a rubber hand without feeling a proprioceptive drift of their real hand towards the artificial limb [100]. Although the proprioceptive effect of the rubber hand illusion was not measured in Reinersmann et al.'s [41] study, this sensory modality has been investigated in the context of an artificial finger illusion discussed below.

Reinersmann et al.'s [41] study suggests intact visuotactile integration in people with CRPS by virtue of a normal rubber hand illusion. On the other hand, a study by Wang et al. [64] suggests that despite impaired proprioception, they can integrate tactile and proprioceptive information and normally experience a multisensory illusion. In their study, people with CRPS were less susceptible to an artificial finger illusion, compared to healthy controls, when only proprioceptive information was available [64]. In the illusion, the hands are positioned one above the other, aligned vertically but some distance apart, and obscured from the participant's view. The index finger of the bottom hand is placed snugly in a pipe, and the index finger of the top hand is placed adjacent to (proprioceptive only condition) or grasping (proprioceptive and tactile condition) an artificial finger. Typically, both of these conditions create an illusion that the hands are closer together in vertical distance than they are in reality [64]. Regardless of which hand (affected or unaffected) was positioned on the top or bottom, this effect was not found in people with CRPS when they were not grasping the artificial finger. Interestingly, people with CRPS did experience the illusion to a similar extent as healthy controls when they received tactile input (i.e., while grasping the artificial finger). This study suggests that although proprioception itself might be altered in CRPS, it can still be integrated with any available tactile information and result in normal performance on a multisensory bodily illusion [64]. The findings of Wang et al. [64] complement those of Reinersmann et al. [41] from the rubber hand illusion with explicit involvement of proprioceptive information and further support the conclusion that people with CRPS have intact multisensory integration.

#### 3.1.5. Summary of Changes in Body Representation

Across the current literature, people with CRPS consistently report symptoms pertaining to altered body representation including asomatognosia, distorted perception of the affected parts of the body, and negative feelings about the affected limb. These findings arise not only from self-report measures, but are in agreement with experimental tests of body representation such as limb laterality recognition [56–60], as well as limb size matching and limb position matching [44, 49, 50, 52–54]. However, manual estimates of body midline were not consistently impaired in people with CRPS [38, 55]. Body representation relies on the dynamic integration of visual, tactile, and proprioceptive information. Broadly speaking, multisensory integration seems to be intact in people with CRPS and thus cannot account for their distorted body representations. The availability of visual cues can improve (but not fully normalize) position sense for the affected limb [52], suggesting that visuoproprioceptive integration is possible. The effects of tool use, the rubber hand illusion, and the artificial finger illusion suggest intact visuotactile [41, 51] and tactile-proprioceptive [64] integration. When whole body movement is concerned [48], auditory-proprioceptive integration can modify subjective perception of the body. Thus, it appears that people with CRPS are able to experience certain body-related multisensory illusions [41, 48, 64] and their performance on proprioceptive tasks improves when congruent input from additional senses is available [52]. Furthermore, people with CRPS are able to update the representation of their body [48], but this process might differ between the affected and nonaffected sides [51]. Greater updating of bodily representations in people with CRPS compared to pain-free individuals suggests that these representations might be less stable in CRPS [51].

Deficits in systematically measured aspects of body representation mostly appear to arise when people with CRPS have to rely on proprioception, and additional sensory cues are either missing (e.g., when positioning the affected limb with eyes closed [52]) or are incongruent with other senses or motor commands (e.g., when visual feedback about the movement is altered [44]). One possible explanation is that proprioceptive information from the affected limb is not reliable. Sometimes proprioception is impaired in the analogous unaffected limb, too [44, 52], which potentially occurs through central mechanisms since in this case the core symptoms of CRPS are not present. There is evidence that we integrate different sensory cues by adaptively making a weighted linear average based on the reliability of each sensory modality [101, 102]. Therefore, disrupted reliability of proprioception in people with CRPS could mean that the weighting of other senses (e.g., vision) is stronger to compensate [102, 103]. Overall, there is consistent evidence that multisensory integration in CRPS is intact. This mechanism is known to contribute to building and updating multimodal body representations [79, 104], and both are governed by similar parietal networks [104–107]. However, neither multisensory nor unisensory representations were directly linked to self-reported body perception disturbance in CRPS [44, 52] (for exceptions, see [41, 48]). Because multisensory integration is intact, it cannot explain the distorted body representation in this population. Therefore, other potentially higher-level mechanisms might contribute to these distortions.

### 3.2. Lateralised Spatial Cognition

In addition to the distortions in body representation discussed in the previous section, many people with CRPS report symptoms resembling the hemispatial neglect syndrome (“neglect”) that can follow a brain lesion. Neglect is an attentional deficit in sensation, movements, and/or representations of the contralesional (usually left) side of body and/or space that cannot be completely attributed to a sensory or motor loss [81]. It most often occurs following lesions to the right inferior parietal lobe and temporoparietal junction [108–111], but can also stem from lesions to other cortical and subcortical areas, such as the mid superior-temporal gyrus, angular gyrus, basal ganglia, and thalamus [112]. Neglect has served as an analogy to describe some of the neuropsychological symptoms found in CRPS. Thus, it is important to consider which aspects of higher cognition are affected in poststroke patients to systematically characterise related deficits in chronic pain patients.  Table 3 summarises examples of deficits shown by people with neglect following brain lesions in different perceptual, motor, and representational modalities; egocentric and allocentric reference frames; and personal, peripersonal, and extrapersonal regions of space (in addition to our use of “reference frames” when distinguishing between egocentric and allocentric encoding of space, “reference frames” can also be used to refer to the distinction between the ways that information in personal, peripersonal, and extrapersonal space is encoded and represented; however, to enable a clear discussion of the overlapping and distinct spatial effects in egocentric/allocentric representations versus personal/peripersonal/extrapersonal representations, in this paper, we reserve the term “reference frames” for the former distinction and “regions of space” for the latter distinction) (for a comprehensive review, see [81]).

Although CRPS is generally not associated with any brain lesions, the unilateral nature of CRPS means that we could expect any cognitive deficits to be predominantly associated with the activity of the hemisphere contralateral to the painful side. However, thus far the evidence for such lateralised manifestations of neuropsychological symptoms in CRPS is not straightforward. In the following sections, we review research regarding spatially lateralised cognitive functions in CRPS, with the primary focus on spatial attention. We aim to discern the discrepancies in the direction of lateralised spatial deficits in CRPS and the particular conditions under which they manifest. Finally, we attempt to integrate the changes in spatial cognition with the evidence of distorted body representation.

#### 3.2.1. Self-Report and Clinically Assessed “Neglect-Like” Symptoms

The first published evidence for systematic spatial biases in CRPS comes from clinical reports [33] and self-administered surveys [36] reporting motor and cognitive changes consistent with neglect of the affected limb. Galer et al. [33] observed “motor neglect” in CRPS, specifically slower movement initiation (hypokinesia), slower movement execution (bradykinesia), decreased movement amplitude (hypometria), and decreased spatial extent of movements performed with the CRPS-affected hand compared to the unaffected one. Further signs of motor neglect in CRPS are patients' reported need for directed attention to move the affected limb and the occurrence of involuntary movements. There are also anecdotal reports of patients who failed to move the CRPS-affected limbs when they were concealed from view despite being convinced that they were performing bilateral arm movements [113]. This phenomenon might be characterised as motor extinction (a deficit of motor production that either worsens or only becomes apparent during bilateral movements [114]), although the authors did not report if performance with the affected limb was better when making unilateral movements under the same conditions. “Cognitive neglect” as defined by Galer and Jensen [36] involves feelings of foreignness and lack of ownership over the affected limb. However, the authors never intended for the term “neglect” to be taken literally in the context of CRPS, and we argue that these symptoms more closely resemble body representation distortion than hemispatial neglect (see Section 3.1.1). Between 17% and 90% of patients with CRPS report motor and/or cognitive “neglect-like” symptoms as defined above [33, 35–40, 42, 62]. Also, the frequency [39] and severity of these self-reported symptoms appear to be greater in CRPS than other pain conditions [35]. Thus, based on this clinical and self-report evidence, it could be argued that people with CRPS present with neuropsychological deficits that resemble hemispatial neglect and related syndromes of body awareness, such as asomatognosia (loss of ownership) [82] and misoplegia (dislike or hatred of the affected limb) [84].

#### 3.2.2. Standard Neuropsychological Tests of Neglect

Following the self-reports of neuropsychological symptoms resembling neglect, some researchers pursued a more objective assessment of these deficits in CRPS by administering classic neurological assessments and pen-and-paper tests that are typically used with brain-injured patients. During confrontation testing, a standard neurological assessment of neglect, patients with poststroke hemispatial neglect typically fail to report seeing or feeling targets presented on the contralesional side, indicating extinction (when the failure is only during bilateral stimulation) or neglect (when the failure is also during unilateral stimulation). Confrontation testing performed by Cohen et al. [71] revealed that only three out of the 22 tested people with CRPS presented with tactile extinction, while Förderreuther et al. [37] did not observe either neglect or extinction in individuals with CRPS. Five of Cohen et al.'s [71] participants, however, showed tactile allochiria (i.e., perceiving unilateral touch only in the analogous contralateral location), which has been reported in several modalities in hemispatial neglect patients [115–118].

One of the classic bedside tests of hemispatial neglect involves dividing a straight horizontal line in half [119]. For example, a patient who has reduced attention to the left side, relative to the right, would ignore the left end of the line and place the bisection mark further to its right side. A deviation from the centre is thus indicative of spatial attention bias. In CRPS, there are only single case studies reporting deviations in classic line bisection performance: one *away* from the affected (right) side of space [65] and one *towards* the affected (left) side of space [53, 54]. Interestingly, Christophe et al. [53] describe that the patient in their study showed a bias towards the affected side when line bisection was performed with either the healthy or affected hand and the line was positioned at body midline. However, positioning the to-be-bisected line in the affected side of space abolished the bias. These single case reports point towards impaired perception of spatial relationships between external objects (allocentric frame of reference) located within reaching distance (i.e., in peripersonal space) [81]. Although the direction of the bias relative to the affected side is inconsistent between the two cases [53, 54, 65], both patients presented with a leftward bias. This appears to be consistent with a third type of abnormal bisection performance that has been reported for people with CRPS, which was found in robot-assisted line bisections performed with the healthy limb [66]. In this group study, independent of the CRPS-affected side of the body, participants' bisections consistently deviated towards the left relative to those of the pain-free controls. These findings resemble an exaggeration of “pseudoneglect.” That is, healthy controls show the consistent leftward deviation on some spatial tasks, which is interpreted as an effect of right-hemisphere dominance in spatial perception [120–122]. Finally, several group studies of people with CRPS have reported no signs of line bisection bias relative to healthy controls [37, 38, 40, 55, 58] when the task was performed with either the affected or unaffected hand. No lateralised impairment was found on other classic bedside tests of neglect, for example, clock drawing, clock reading, rod orientation, Kohs blocks, or block tapping [38].

Overall, the performance of people with CRPS on confrontation testing and standard neuropsychological tests does not provide sufficient support for the hypothesis that CRPS involves neglect of the affected limb or side of space. Some findings even suggest the opposite direction of spatial bias or exaggerated “pseudoneglect.” The inconsistence between the normal performance of people with CRPS on classic bedside tests of neglect in most studies, despite the high percentage of self-reported “neglect-like” symptoms in large sample studies (e.g. [35, 36, 39, 42]), might stem from the differences between what these two types of measures entail. That is, the questionnaire about “neglect-like” symptoms measures asomatognosia and motor aspect of neglect, whereas classic bedside tests of neglect primarily measure its perceptual aspect (although they usually require motor responses, too). Another possibility is that classic neglect tests are not sufficiently sensitive to reveal the subtle neuropsychological changes in CRPS, given that classic pen-and-paper tests of neglect were developed to test people who suffered brain lesions, and neuropsychological changes in CRPS are likely to develop because of less overt structural and/or functional changes.

#### 3.2.3. Sensitive Measures of Lateralised Cognitive Functions

Inconsistent findings regarding the spatial bias in people with CRPS led some researchers to measure lateralised spatial cognition using methods that are more sensitive. Substantial research on lateralised spatial deficits in brain-lesioned patients and healthy controls has revealed that better precision and sensitivity of assessment can be achieved through experimental manipulation of the properties of the stimuli used to measure attention, spatial representations, and motor control and by altering the conditions under which these tasks are performed. We present the evidence available from several sensitive measures of lateralised changes: the subjective body midline task, temporal order judgements, mental number line bisection, and tests of spatially defined motor control. Through these tasks, researchers have found evidence for biases in people with CRPS in the following domains of spatial cognition: the egocentric frame of reference, tactile spatial attention in personal space, visual spatial attention in personal and peripersonal space, the internal representation of space, and spatially defined motor control.

*(1) Subjective Body Midline*. In the visual subjective body midline judgement task (or “visual straight ahead”), participants verbally indicate when a light moving horizontally from one side of extrapersonal space to the other crosses the point that is directly in front of the middle of their body. When performed in the dark, with no other visual cues available, the task is thought to measure any lateral shift of the egocentric frame of reference, defined as the coding of the location of external objects in relation to one's own body midline [68, 123, 124]. Multiple studies reported a deviation of subjective body midline towards the affected side of space in people with CRPS compared to healthy and pain controls when judged in a darkened room (median deviation from objective midline ranging from 0.59° to 5.13° [53, 54, 67–69]). The people with CRPS showed no bias in body midline under illuminated conditions, when it is possible to make use of the allocentric frame of reference (external cues). This suggests that if people with CRPS have a distorted subjective body midline, it affects only the representation of external space in relation to their own body. Christophe et al. [53] also demonstrated a distance-based dissociation in one patient who showed a significant deviation towards the affected side when stimuli were presented at two-meter distance from the trunk (similar to other studies cited in this section) but not at one meter. The spatial bias of egocentric frame of reference towards the affected side is consistent with an overrepresentation of the affected relative to unaffected side of space. In contrast to the above findings, Reinersmann et al. [40] found that their participants with CRPS made subjective body midline judgements that were biased further towards the left than healthy and pain controls (0.7° vs. 0.1° and 0.09°), irrespective of which side of the body was affected. This pattern can be interpreted as exaggerated “pseudoneglect,” consistent with the previously discussed findings from the robotic line bisection study by Verfaille et al. [66], and could be due to disruption of right hemisphere cortical networks involved in spatial processing. Visual straight ahead biases, both towards the affected side and towards the left side, suggest that people with CRPS can have problems with combining external visual information with their subjective body midline. Yet other authors demonstrated that people with CRPS showed no bias when judging their body midline using the visual straight ahead task [42, 55]. Thus, it appears that any shifts of egocentric frame of reference are subject to high individual variability, because these effects do not always replicate.

*(2) Temporal Order Judgement*. According to the law of prior entry, attended stimuli are perceived before unattended ones [125, 126]. This principle forms the basis of temporal order judgement (TOJ) tasks. In TOJ procedures, the participant is presented with pairs of identical stimuli, one on each side of space, with different onsets. They report the temporal order of the two stimuli, that is, which occurred first/second. The pattern of left-right responses across different stimulus onsets indicates whether participant's attention is shifted towards one side of space relative to the other. The TOJ task is a sensitive measure of lateralised spatial attention, that is, the distribution of covert attention in one side of space relative to the other.

On tactile TOJ tasks, people with CRPS exhibited reduced attention to tactile stimulation applied to the affected limb (i.e., touch on the affected limb had to occur ~17-27 ms before touch on the unaffected limb for the two stimuli to be perceived as simultaneous [58, 72, 73]; however, Filbrich et al. [74] failed to replicate this effect). When the limbs were crossed, their performance indicated inattention to the unaffected hand, now located in the affected side of space (touch had to occur ~18 ms earlier than on the affected hand in the unaffected side of space [73]). CRPS participants exhibited the same pattern of attention bias both with and without visual feedback about the limbs' position [72]. Tactile stimulation inherently involves body-relevant information; thus, it would seem that the tactile TOJs should rely on a personal frame of reference. However, it appears that those judgements at the same time rely on the current location of the body parts in peripersonal space.

The tactile attention bias away from the affected side also extends to TOJs about visual stimuli presented near [74] or on the surface of the patients' hands and on a blank board in near space [45] (with magnitude of ~14-25 ms). In accord with Moseley et al. [73], the authors concluded that visual attention bias in CRPS is space-based, because it was observed regardless of the involvement of the body. However, Bultitude et al. [45] also found no lateral shift of visual attention when the limbs were crossed such that the affected limb was located in the unaffected side of space. This suggests that people with CRPS had a deviation in attention both away from the affected side and from the affected limb (regardless of where it was located), which cancelled each other out when the limbs onto which the visual stimuli were presented were crossed.

Despite evidence for spatial attention bias from TOJs, these deficits do not seem to affect all aspects of visual spatial attention in CRPS. Filippopulos et al. [75] argued that attention deficits in CRPS do not involve allocation of visual attention, as they failed to find any delay of orienting saccades to cued and noncued visual targets presented in either hemifield. Similarly, no spatial bias away from the affected side of space was found on a computerised task measuring simple reaction times to visual stimuli [38]. The contrasting results on the TOJ tasks and these other computerised tasks might be because of the different regions of space involved, since computer monitors are invariably placed within the participant's extrapersonal space (e.g., at a distance of 60 cm) rather than personal or peripersonal space.

In summary, the results on sensitive tests of spatial cognition in people with CRPS tend to indicate that judgements of their subjective body midline are biased *towards* the affected side, that is, in the direction opposite to what would be expected based on their self-reported “neglect” of the affected limb. Yet, TOJs of tactile and visual stimuli tend to be systematically biased *away* from the affected side of space, and problems with attention allocation [67] cannot explain this bias. Given that both visual and tactile TOJs were affected [45, 58, 72, 73], attention biases in CRPS might be supramodal. On the other hand, when the same individuals were tested on TOJs in multiple modalities, one study found that they only presented with visual but not tactile biases [74] and another study found that they only presented with tactile but not auditory biases [58]. Similar dissociations between sensory modalities can also be found in neglect after brain injury [127].

*(3) Mental Number Line Bisection*. Analogous to the conventional line bisection task that involves the allocentric frame of reference, the mental number line bisection task is thought to involve the “bisection” of the internal representation of space. It is considered to be an implicit measure of mental spatial representations [128] and is independent of motor abilities. In mental number line bisection, participants verbally indicate, without calculating, the number that is halfway between a given pair of numbers. Because the number line is internally represented from left to right [129–131], a bias towards the higher numbers would be equivalent to a rightward spatial bias, as has been demonstrated in hemispatial neglect [128, 132–134]. Midpoint number judgements in CRPS were found to deviate away from the affected side compared to healthy controls [67]. The opposite direction of such a bias was observed in a single case of CRPS of the left limb [53, 54], who also presented with a consistent leftward bias on a range of other spatial tasks. Despite this exception, the group study suggests that inattention to the affected side of personal and peripersonal space exhibited by people with CRPS also affects the internal representation of space. In contrast to personal and peripersonal space, mental number line bisection does not rely on bodily information about the affected limb and its position in external space or the visual representation of the affected side of space. Therefore, biased mental number line bisection suggests a generalized distortion of spatial representations in CRPS, which could potentially occur via shared higher-order mechanisms.

*(4) Spatially Defined Motor Control*. Following the early clinical and self-reports of motor “neglect-like” symptoms [33, 36], several studies also tested for spatially lateralised deficits in movements using sensitive experimental measures. Contrary to the motor neglect hypothesis, people with CRPS did not show any signs of neglect or extinction on behavioural motor tasks such as finger tapping when performed with one or both hands, in normal posture or with the hands crossed such that the affected limb was located in the unaffected side of space and vice versa, or with or without visual feedback [53, 55]. Similarly, there was no asymmetry (i.e., extinction) in hand movement patterns while performing a bimanual circle drawing task measuring motor accuracy [55]. The performance of people with CRPS on both the tapping and circle drawing tasks did not differ from healthy controls. Another study with a larger sample size (13 vs. 7) and a slightly different measure of finger tapping found worse motor accuracy and coordination on circle drawing and button pressing tasks when using the affected limb compared to the unaffected limb, regardless of the side of space in which patients performed the tasks. Importantly, the people with CRPS also showed similar deficits when the tasks were performed on the affected compared to unaffected side of space with the unaffected hand [76]. Thus, there appear to be spatially defined motor deficits in CRPS (that is, deficits modulated by where the movements are performed relative to body midline). It is not possible to ascertain whether the asymmetries between the affected and unaffected limbs and sides of space reported in people with CRPS were greater than normal, because there was no control sample [76]. Nonetheless, the findings of this study are consistent with self-reported “neglect-like” symptoms, which primarily entail movement difficulties [33, 36]. However, another perspective that we will now outline is that motor deficits in CRPS arise from decreased use of the affected limb rather than attention bias [3].

Punt et al. [3] proposed a learning-based account for motor deficits in CRPS framed as nonuse of the affected limb. Learned nonuse manifests as motor difficulties greater than expected based on actual physical constraints or as a difference between what the patients do spontaneously and what they are able to do in clinical examination. This could explain why motor “neglect-like” symptoms are reported by the people with CRPS but not necessarily apparent upon experimental testing [55]. After a stroke, learned nonuse develops through operant conditioning and can affect the entire contralesional side of the body. Punt et al. [3] argued that in CRPS learned nonuse is normally limb-specific rather than involving the entirety of one hemibody and could manifest in protective behaviours (e.g., guarding and holding an affected hand close to the chest). However, despite these differences in the manifestation of learned nonuse in CRPS compared to stroke, its progression is thought to follow a similar pathway [3]. Limb trauma is followed by enforced immobility, leading to poor coordination and dexterity, which result in less frequent attempts to move. Movement is additionally suppressed by pain and fear avoidance behaviours [135]. At the same time, compensatory movements of the unaffected limb are developed and reinforced. These changes can alter cognitive and cortical representation of the CRPS-affected limb [3]. For instance, primary somatosensory and motor cortical representations of the affected hand were found to be smaller (compared to the unaffected hand and to representations of healthy controls) [85, 86, 136–141], consistent with underutilization, while the sensory map of the unaffected hand was found to be enlarged [142], consistent with compensatory use (although these findings have recently been disputed [143]).

In contrast to the framework of motor neglect that attributes spatially defined motor impairments to attentional deficits, the proposal of Punt et al. [3] explains motor control deficits using a learning-based theoretical account. In an attempt to dissociate these two possible explanations of visuomotor deficits in CRPS, Verfaille et al. [66] analysed goal-directed movements of the unaffected limb to bisect horizontal lines in both sides of space. Contrary to the neglect framework, the bisections of participants with CRPS did not show a bias in relation to the affected side nor depending on in which side of space the bisections occurred. Nonetheless, they showed a significant leftward bias, consistent with exaggerated “pseudoneglect.” This finding opposes the learned nonuse account, because the participants performed the bisections with the unaffected limbs. To disentangle the account of motor neglect, future research could investigate if there are any signs of directional hypokinesia or bradykinesia in CRPS. If people with CRPS show performance asymmetries analogous to that of patients with hemispatial neglect after brain injury, they should have slower initiation or execution of movements directed towards the affected side of space compared to movements directed towards the unaffected side of space, even when the unaffected hand is used. All movements in Verfaille et al.'s [66] study were directed towards the CRPS-affected side of space, and thus, it was not possible for their study to discern directional “neglect-like” motor changes. Nonetheless, even based on the evidence available thus far, attention-based and learning-based explanations are not mutually exclusive and some changes in motor control in CRPS could arise from a combination of both.

Although Punt et al. [3] sought to separate perceptual and motor aspects of neglect, we propose that their learned nonuse hypothesis can also provide a basis for explaining how perceptual spatial biases could arise in CRPS. Previous studies involving amputees and healthy participants with limb immobilization provide evidence in favour of action-driven spatial representations (see also [144]). Specifically, upper limb amputees were found to “neglect” the side of near (but not far) space corresponding to their missing arm [145], and in healthy participants, experimental cast immobilization of one arm led to shrinkage of its peripersonal space [146]. These findings suggest that lack of limb action can change the representation of space surrounding that limb. Because of decreased mobility of the affected limb, people with CRPS perform fewer movements in the affected side of near space. We hypothesise that this could give rise to changes in the cognitive representation of space. Underrepresentation of the CRPS-affected side of space could potentially hinder the ability to perform motor tasks on that side, in line with spatially defined deficits in motor accuracy and coordination found in people with CRPS [76]. It could also contribute to reduced attention to that side of space demonstrated in TOJ studies [45, 58, 72–74].

#### 3.2.4. Summary of Changes in Lateralised Spatial Cognition and Potential Mechanisms

Overall, research suggests that people with CRPS might present with neuropsychological deficits resembling hemispatial neglect that can follow a stroke. However, the evidence is not consistent. Researchers have rarely found lateralised spatial biases using standard bedside measures of neglect or using sensitive measures such as saccades and reaction times to visual targets, auditory TOJs, and some experimental measures of motor performance. Other sensitive tests of perceptual (visual or tactile TOJs) and representational (mental representation of space) changes have revealed lateralised deficits in spatial cognition consistent with a bias *away from* the CRPS-affected side of the body and/or space. Additionally, other findings from visual subjective midline judgements point to a shift of egocentric frame of reference *towards* the affected side in CRPS, thus in the direction opposite to what would be expected for neglect of the affected side. The opposing biases *away from* the affected side of space in TOJ tasks and *towards* the affected side in visual subjective body midline cannot be explained by the different modalities that are tested in these tasks, because TOJs were biased in the visual domain. We consider two possible explanations for these opposing biases: the dissociation between near and far regions of space and the distinct functional aspects of peripersonal space (defensive and goal-directed).

*(1) Near Space versus Far Space*. The different regions of space in which participants perform the TOJs and subjective body midline judgements could potentially account for the inconsistent biases shown by people with CRPS on these tasks. The studies using visual subjective body midline judgements in CRPS presented stimuli in far/extrapersonal space (generally two meters away from the trunk). The studies using TOJs, on the other hand, presented stimuli in either personal space (e.g., tactile TOJ, visual TOJ when stimuli are presented on body surface) or near/peripersonal space (e.g., visual TOJ when stimuli are presented on a blank board within arms' reach or immediately next to the hands). Like perceptual TOJs, the internal representation of space (as measured through mental number line bisections) is also biased away from the affected side. Dissociations between distinct regions of space have been found in some poststroke hemispatial neglect patients, where attention deficits manifested either exclusively in their personal space [147], near/peripersonal space [148], far/extrapersonal space [149, 150], or internal representation of space [132, 151]. Although rare, there are reports of individual patients with poststroke neglect [152–155] who show opposite directions of bias on different tasks, as also reported in Sumitani et al.'s [67] CRPS study (opposing biases in subjective body midline and mental number line bisection).

*(2) Defensive versus Goal-Directed Space*. In the above statement, we have suggested a possible explanation for the inconsistent biases shown by people with CRPS on TOJ and visual straight ahead tasks based on known cortical dissociations between the representation of near and far space identified through research on brain-lesioned patients. However, given that people with CRPS typically do not have any history of brain damage, it could be more meaningful to consider potential cognitive mechanisms that might better account for the different results on this task. Peripersonal space is thought to dissociate into two representations according to distinct functions: for preparing defensive responses (defensive peripersonal space) and for preparing actions (goal-directed peripersonal space) [156]. Furthermore, Bufacchi and Iannetti [157] argue that peripersonal space cannot be defined in terms of fixed boundaries around the body (or body part), but its extent is rather graded and dynamically changing according to the action being performed and the proximity or valence of external information. Thus, we speculate that different dynamic changes to goal-directed and defensive peripersonal space specific to the affected extremity [158] might explain the contrasting biases that have been reported in people with CRPS at different distances from the body. Reduced activity of the affected limb [3], resulting in fewer interactions with the affected side of goal-directed peripersonal space, could reduce visuospatial processing near the body in the affected compared to unaffected side. For example, Makin et al. [145] found that visuospatial processing of amputees favoured their intact side when stimuli were presented at a distance of 50 cm. The biased TOJs in people with CRPS were observed within the same distance (see also [158] for a review of how peripersonal space is shaped by action and integration of multisensory information from the body and the environment). In contrast, it has been shown in healthy participants that approaching, threatening stimuli can extend peripersonal space in such a way that is sensitive to the trajectory of the threat [159, 160]. No studies have measured the dimensions of the affected side of defensive peripersonal space in CRPS. However, we suggest that it could be enlarged due to heightened hypervigilance to threat, as has been reported for the representation corresponding to the affected area in trigeminal neuralgia [161]. This could explain why people with CRPS showed greater tool use-dependent updating of peripersonal space than controls [51], which could indicate that their spatial representations are less stable. It is conceivable that such a heighted defensive awareness to stimuli that are potentially threatening to the CRPS-affected limb (due to allodynia and hyperalgesia) could drive a bias towards the affected side in extrapersonal space. This might particularly be the case for dynamically moving stimuli such as those used in the visual subjective midline task. This speculation should, however, consider that the visual subjective body midline in CRPS has typically been assessed at two-meter distance from the trunk, which is beyond the extent of peripersonal space normally reported in healthy participants (80-90 cm [162]). Body midline judgements made at one meter were not biased in a case of CRPS [53], similar to a group study that reported no bias on visual TOJs for stimuli presented 90 cm from the trunk [74]. However, thus far, no studies have mapped the extent of defensive peripersonal space in people with CRPS in the context of threatening and/or dynamically approaching stimuli (note that the TOJ stimuli appeared in a fixed distance from the participant's body). Spatial representations can be dynamically changing depending on the conditions and the meaning of the testing stimuli. Therefore, an enlarged defensive yet diminished goal-directed peripersonal space representation of the affected side could still account for the seemingly contradictory findings of attention bias in CRPS.

On balance, the discussed findings suggest that CRPS is associated with contrasting alterations in spatial attention, representations of space, and spatially defined motor control. The neuropsychological changes in these domains are observed in different modalities (visual and tactile) and different regions of space (personal, peripersonal, extrapersonal, and representational). The existing evidence cannot fully account for the conflicting directions of the spatial biases that have been reported (towards or away from the CRPS-affected side). Hypothetically, some of the contrasting patterns of performance in the spatial tasks could be explained by hypervigilance to approaching stimuli within the affected side of extrapersonal or defensive peripersonal space, simultaneous to “neglect” of the affected side of personal and goal-directed peripersonal space stemming from learned nonuse.

#### 3.2.5. Overlap of Body Perception Distortion and “Neglect-Like” Symptoms

Thus far, we separately reviewed evidence for body perception disturbances and deficits in lateralised spatial cognition in CRPS. However, these two cognitive functions are inherently linked (e.g., spatial representations are anchored in the represented location of the body [158, 163]), and neuropsychological changes in them often present simultaneously [45, 58]. Somatosensory, motor, and body representation distortions are largely confined to the CRPS-affected limb (although bilateral and hemisensory deficits have also been reported, e.g., [23, 26, 52, 164]); thus, they can be considered primarily lateralised. This is comparable to the changes in spatial cognition discussed so far, which most often take the CRPS-affected side as a point of reference. Whether problems with body representation and attentional orienting are truly dissociable in CRPS remains uncertain. For instance, Reid et al. [58] suggested that interactions between spatial attention and processing of body-relevant information (e.g., seeing the limbs) might exacerbate usually subtle lateralised spatial changes by evoking distorted body representation.

*(1) The “Somatospatial Inattention” Hypothesis*. Some spatial biases might only manifest when the body is directly involved in the task at hand, demonstrating an overlap of the cognitive changes in body representation and spatial attention. When directly investigating these interactions, Reid et al. [58] found a deviation away from the affected side in people with CRPS when line bisections were performed on the surface of their hands but not when performed on paper. This perceptual bias was space-dependent, because it was present not only on the affected limb but also on the healthy limb when placed in the affected side of space. Participants with CRPS exhibited a similar deviation away from the affected side when they bisected the length of their affected hand and forearm [58]. Interaction between spatial bias and body representation was also demonstrated by difficulties with recognising the laterality of body parts specifically when they were presented in the affected hemifield [58]. Based on this evidence, and the previously found attention bias away from the affected side on tactile TOJs, Reid et al. [58] proposed that the disruption of spatial processing in CRPS specifically involves problems with integrating spatial information with body representation, a phenomenon they called “somatospatial inattention.” This hypothesis was partially supported by Filbrich et al. [74], who found a significant attention bias in visual TOJs only when patients' hands were positioned close to the visual stimuli in near space, but not when the hands were out of sight, close to the trunk. Deviated visual subjective body midline in CRPS [67–70] is also somewhat in agreement with this hypothesis, since this measure requires integrating body midline with the external visuospatial reference frame. However, in this case the performance of people with CRPS is consistent with overrepresentation of the affected side rather than inattention. Furthermore, the proposed “somatospatial inattention” does not fully account for all spatial attention biases found in CRPS, because significant deviation away from the affected side was also observed in visual TOJs for stimuli that did not involve and were not near to any body parts [45].

*(2) Proposed Mechanisms of Interactions between Bodily and Spatial Representations*. We suggest that there are two hypothetical mechanisms through which body representation disturbances might drive attentional biases even when body parts are not directly involved in the spatial tasks: reduced ownership and increased perceived size of the CRPS-affected limb. More generally, body representation forms the basis for spatial cognition [158, 165]. In CRPS, reduced awareness and ownership of the painful limb could contribute to inattention to the affected side. For example, the severity of body perception disturbance was found to predict the magnitude of spatial attention bias away from the affected side in people with CRPS [45]. Furthermore, a perceived increased size of the affected extremity [49] could conversely drive hyperattention to that side.

Peripheral CRPS symptoms in the affected limb might offer an additional explanation of how body-related disturbances could drive attentional biases. First, it has been suggested that the bias in visual subjective body midline judgements towards the CRPS-affected side is due to an exaggerated somatosensory input from the painful limb [68, 166]. Second, CRPS signs can manifest as a combination of sensory gain (e.g., pain and hyperalgesia) and sensory loss (e.g., hypoesthesia) [167]. Thus, suppression of some types of somatosensory input could potentially explain tactile inattention to the affected limb (e.g., on TOJ tasks when the hands are uncrossed). Third, mechanical constraints related to motor symptoms of CRPS can trigger underutilization of the affected limb [3]. As we argued in Section *(4) Spatially Defined Motor Control*, such underutilization could lead to space-based inattention, because fewer movements performed in the affected side of space would drive asymmetries in spatial representations. Although these peripheral somatosensory and motor abnormalities are not equivalent to distorted body representation, this representation is generated and continuously updated based on multimodal sensory input and motor feedback during action [79, 80, 158, 165]. Therefore, the peripheral (somatosensory and motor) and central (body representation) mechanisms could serve as complementary explanations of how body-related information could exacerbate spatial biases, even when that information is not directly relevant to the task. Nonetheless, direct empirical evidence for how body representation, somatosensory, and motor disturbances might shape spatial processing in CRPS is limited, and it remains unclear why the attention bias is sometimes found to be shifted away and sometimes towards the CRPS-affected side.

In conclusion, people with CRPS show several changes to lateralised spatial cognition. These share many similarities with hemispatial neglect, yet there are also several differences. Although the abovementioned aspects of body representation disturbance might relate to lateralised attention deficits, they should not be treated synonymously (i.e., as “neglect-like” symptoms). A distinction between the two concepts can help to avoid theoretical, terminological, and mechanistic confusion in research.

### 3.3. Non-Spatially-Lateralised Cognition

In addition to changes in body representation and lateralised spatial cognition reviewed thus far, people with CRPS can also present with cognitive deficits that are not lateralised with respect to the affected side of the body or space. In this section, we discuss non-lateralised cognitive processes that comprise aspects of both spatial and nonspatial cognition. Examples of potentially affected aspects of non-spatially-lateralised spatial cognition include spatial orientation, memory for spatial locations, visuospatial exploration and coordination, constructional abilities, and knowledge about the orientation and order of objects, letters, or numbers. Examples of potentially affected aspects of non-spatially-lateralised nonspatial cognition include numerical and language processing, recognition of objects and faces, imitating complex movements, generalised attention, working memory, and executive function. Broadly speaking, these can be broken into cognitive functions that have been associated with the parietal lobe and executive functions, memory, and language.

#### 3.3.1. Parietal Functions

Comprehensive standard neuropsychological assessments of people with CRPS revealed no systematic abnormalities in spatial orientation, visual exploration, constructional abilities, spatial memory, or visuospatial coordination on a group level, compared to healthy and pain controls [38]. However, Cohen et al. [71] assembled a custom battery of standard neuropsychological tests to assess functions specifically associated with the parietal lobe. They found that 68% of their tested participants with CRPS showed one or more deficit in the ability to recognise objects by touch (astereognosia), identify the fingers of the hand (finger agnosia; see also [37, 77]), identify numbers outlined on the surface of the hand (dysgraphaesthesia), draw objects (constructional apraxia), comprehend arithmetic (dyscalculia), write (dysgraphia), repeat speech (conductional dysphasia), differentiate between the left and the right side of the body, and/or imitate gestures or tool use (ideomotor apraxia). Deficits like these all typically occur after parietal lobe lesions [168]. However, the assessed individuals with CRPS had never sustained brain injury that could account for these deficits (confirmed by normal MRI scans in 12 out of 22 patients) and had not had any cognitive difficulties prior to the onset of CRPS symptoms (corroborated by their families). None of the healthy control participants tested on a shortened version of the same battery presented with any neuropsychological deficits, suggesting that these symptoms could be due to CRPS-related functional cortical reorganization of the parietal networks. Although tested on both upper limbs, the abnormalities on the manual and tactile/haptic tests were only present on the affected side of the body of participants with CRPS. This means that some of the observed deficits could be attributed to peripheral sensory loss or motor impairment. However, 27% of patients with lower limb CRPS also presented with behavioural deficits despite being tested on their unaffected upper limbs [71]. Therefore, it is likely that at least some of the reported changes are due to cortical reorganization that is driven by parietal changes.

There are also reports from this and other studies of individual people with CRPS who presented with more unusual and severe non-spatially-lateralised deficits. Cohen et al. [71] reported cases of horizontal inversion of individual letters and words, and inverted ordering of letters or numbers, in spontaneous writing (resembling a form of dysgraphia [169]), although people with CRPS did not show any impairment of letter orientation recognition in a different study [58]. These deficits were apparent when patients used their affected limb and in one patient bilaterally. Robinson et al. [65] also presented a case of a right upper limb CRPS patient with no history of brain injury who exhibited mirror reversal in writing single words with his unaffected hand and in reading single letters. Mirror writing is rare, but can follow various focal lesions to the left hemisphere [170, 171]: the hemisphere contralateral to this patient's CRPS-affected hand. The same patient also presented with severely impaired face perception (i.e., prosopagnosia, a neuropsychological symptom that can occur following a lesion to fusiform gyrus on the ventral surface of the temporal lobe [172]) that had not been present prior to the development of CRPS. Despite being able to visually recognise and name objects, the patient failed to recognise if objects were in the upright orientation and he copied objects into inverted orientations. Orientation agnosia is most commonly found in patients with lesions to the posterior parietal cortex [173–175].

The studies directly assessing parietal lobe function in CRPS thus far have had relatively small sample sizes and usually lack pain or age-matched control groups (although unspecified control samples were tested on most of the tasks in Cohen et al.'s study [71]). Therefore, it is difficult to estimate the real prevalence of the symptoms discussed above in CRPS. An exception is a study by Kolb et al. [38], who tested for several neuropsychological symptoms linked to parietal function. In this study, people with CRPS on average did not present with any abnormalities that would be consistent with parietal dysfunction. However, the authors did not report individual cases and for some measures did not specify which hand was tested (for instance, Cohen et al.'s [71] patients were not impaired when using their unaffected hand). We cannot argue that the neuropsychological changes discussed in this section are common in CRPS population, because they were observed only in a proportion of patients or in single cases (see Table 2). Nevertheless, reports of deficits in CRPS that are typical of patients with temporal and parietal lesions suggest a disruption of visuospatial functions that could be due to functional cortical reorganization in these areas.

#### 3.3.2. Executive Functions, Memory, and Language

Although there is evidence for biased *spatial* attention in people with CRPS, not all aspects of attention appear to be affected in this population. Specifically, no differences between people with CRPS, healthy controls, and pain controls were found on measures of alertness (response readiness) and working memory [62]. People with CRPS did, however, have poor temporal acuity when making spatial judgements. Specifically, in a visual TOJ task, they needed larger intervals between the two stimuli to reliably indicate their order of presentation [45]. In another, large sample study (*N* = 137), 42% of people with CRPS presented with mild dysexecutive syndrome (relative to age- and education-matched normative data), including impaired performance on working memory and verbal fluency tests [78]. Twenty-three percent of people with CRPS showed global cognitive processing impairments. Besides executive deficits, they also demonstrated impaired naming and declarative memory [78]. Executive, naming, and memory deficits are consistent with pathology of the frontal lobes. Together with the deficits in general (non-lateralised) spatial cognition, problems with language processing also suggest changes to parietal function in CRPS.

#### 3.3.3. Summary of Non-Spatially-Lateralised Cognitive Changes

In summary, people with CRPS can present with non-spatially-lateralised deficits in higher cognition that resemble impairments found in neurological conditions other than hemispatial neglect. Findings from standard neuropsychological test batteries are still mixed; however, some individuals with CRPS present with neuropsychological symptoms like those shown by patients with lesions to the parietal lobe (e.g., astereognosia, finger agnosia, or constructional apraxia) and/or temporal lobe (e.g., mirror reversal of writing, object orientation agnosia, or prosopagnosia). These unusual symptoms appear to affect only a subset of people with CRPS, yet they demonstrate that changes in visuospatial functions are not limited to lateralised spatial processing biases. Furthermore, people with CRPS can also present with features of dysexecutive syndrome and some language processing difficulties that are typical of frontal and parietal lobe pathology. Hemispatial neglect most often occurs after a lesion to temporoparietal regions of the right hemisphere [108], which would be expected to disrupt other neuropsychological functions that depend on these networks. Thus, non-spatially-lateralised deficits can also cooccur with neglect. Such changes include impaired sustained attention, impaired selective attention, a tendency to favour local features over global configurations, and deficits in spatial working memory [112] (for reviews, see [176, 177]). In addition, these symptoms are not diagnostic features of neglect. This combined evidence suggests that the neglect framework is useful but not sufficient for characterising the breadth of neuropsychological changes in CRPS. Instead, the disruption of parietal function and/or cortical networks involving the parietal lobe appears to be a better candidate.

Although there is no direct neuroimaging evidence linking parietal cortex to cognitive deficits in CRPS, several studies on sensory and motor function reported altered patterns of activation in parietal regions. For instance, tactile stimulation of the fingers of both hands resulted in weaker superior [77] and inferior parietal lobe evoked responses [140] in people with CRPS compared to healthy controls. Furthermore, relative to healthy people, individuals with CRPS showed greater activation of the inferior parietal lobe during movement (relative to rest) of the affected compared to unaffected hand [178] and when they were observing hand movements (relative static hands) [179]. Finally, another study reported reduced grey matter volume in the inferior parietal lobe in early-stage (less than 10 months) CRPS, compared to healthy controls [30]. These parietal regions have been linked to the perception of space and limb location in other studies [180, 181], which supports the conclusion that functional and/or structural reorganization of parietal networks might be associated with neuropsychological symptoms in CRPS. However, further studies are necessary to test this hypothesis and identify the neural underpinnings of these cognitive changes.

## 4. Clinical Relevance of Neuropsychological Changes in CRPS

In the following sections, we will discuss the clinical significance of aberrant changes in higher cognitive functions in CRPS. Their interactions and relationships with clinical signs of the disorder reflect the role of the neuropsychological changes in the manifestation of CRPS. They can also inform the treatment approaches targeting these higher cognitive changes to improve the clinical outcomes.

### 4.1. Supraspinal Modulation of Sensory, Motor, and Autonomic Functions

Although this review primarily focuses on higher-level cognition, here, we provide examples of cortical modulation of low-level sensory, autonomic, and motor functions in CRPS (Table 4), relevant to understanding the higher-order central mechanisms of clinical signs of this condition. Previous research suggests that resting or seeing the affected limb in the unaffected side of space can normalize the temperature of that limb [72, 182] (although this effect is not always found [51]). Furthermore, manipulating the perceived size of CRPS-affected hands can modulate movement-related pain intensity and swelling [183]. Sensory conflicts, such as viewing ambiguous visual stimuli, can increase pain and induce other sensory disturbances, dystonic reactions, and asymmetric autonomic response [184, 185]. Sensory disturbances associated with increased pain can also be triggered by sensory-motor conflicts [186]. Heightened susceptibility to such conflicts suggests that CRPS-related sensory impairments might extend beyond the cortical networks related to sensory-motor processing of the affected body parts. Specifically, they can arise from processing visual objects [184, 185] or sound [187] unrelated to the body or during movements of the unaffected arm [186]. People with CRPS also presented with abnormal sensations in the CRPS-affected limb evoked without actual somatosensory stimulation, solely by creating a visual illusion of the affected limb being touched [188]. Overall, the many examples of relief or worsening of symptoms by spatial or multisensory manipulations support the notion that sensory and autonomic abnormalities in CRPS cannot be fully accounted for by peripheral mechanisms and suggest an involvement of supraspinal cortical mechanisms in generating or aggravating physical symptoms of CRPS.

### 4.2. Neuropsychological Symptoms Related to Pain Intensity

Interrelationships between the changes in higher cognitive functions and clinical signs of CRPS further demonstrate the involvement of central mechanisms in the manifestation of the syndrome. For example, higher pain intensity was associated with greater body perception disturbance, longer time taken to recognise the laterality of images of the affected limb, and more impaired sense of limb movement [44, 47, 57, 60]. People with CRPS also reported increased pain intensity while completing the limb laterality recognition task, which was greater in higher cognitive load conditions (i.e., when limbs were presented for shorter time) [63]. Finally, the severity of spatially modulated motor deficits [76], self-reported “neglect-like” symptoms [42], and magnitude of spatial attention bias [58, 72, 73] were related to more intense pain, although several studies reported finding no such relationships [39, 40, 45, 74]. Nevertheless, self-reported “neglect-like” symptoms might have important prognostic value and contribute to the maintenance of CRPS, because they predict pain outcomes six months later in chronic CRPS [42]. The existing behavioural evidence cannot ascertain whether neuropsychological symptoms are primary or secondary to clinical signs of CRPS. However, the reported relationships between these outcomes suggest that cognitive and behavioural interventions targeting changes in processing conflicting information, body representation, and lateralized spatial function have a potential to improve clinical outcomes in CRPS and other pain conditions.

### 4.3. Are Neuropsychological Symptoms Specific to CRPS?

One outstanding question is to what extent the neuropsychological symptoms that we have reported here are unique to CRPS. Of those neuropsychological changes we have discussed, space- and body-related neurocognitive phenomena often relate to clinical symptoms of CRPS and might be specific to this pain syndrome. The lateral shift of subjective body midline [40, 70], overestimation of the size of the affected limbs [49], referred somatosensation from the healthy to the affected limb under mirror visual feedback [188], and sensory disturbances and increased pain due to viewing conflicting visual stimuli [185] seem to be unique to CRPS. This is because they were not found in control patients with other pain disorders who participated in the same studies.

However, changes in body representation [190], spatial representations [161], auditory perception [191], tactile acuity [192], and proprioception [190] can also be present in other chronic pain conditions. For instance, despite being slower than healthy participants in recognising hand laterality, when the performance of participants with CRPS was directly compared to those with phantom limb pain [62] or other non-CRPS upper limb pain [40], there were no differences compared to these groups. Self-reported “neglect-like” symptoms were also found in other chronic pain conditions, particularly upper limb pain [33, 36–38, 40, 62] (although see [35]). Thus, some deficits in body representation and lateralised spatial cognition appear to be present in lateralised chronic pain conditions other that CRPS. Altered body representation was also observed in widespread pain (fibromyalgia) and chronic back pain (for a review, see [190]). People with fibromyalgia also reported similar experiences during sensory-motor conflict as individuals with CRPS [186]. It is thus possible that the above changes in body representation are common features of a group of related chronic pain conditions.

Certain cognitive changes might be associated with chronic pain more generally, regardless of its site and origin. For instance, deficits in working memory, verbal learning and memory, and nonlateralised attention have been found in people with chronic pain other than CRPS [95, 193]. A comprehensive literature review by Hart et al. [193] concluded that attentional capacity, processing speed, and psychomotor speed are commonly affected in people with chronic pain (without a history of brain injury) compared to healthy controls. The severity of their cognitive deficits has often been associated with reported pain intensity, and most studies ruled out the effect of medication on the participants' performance. Even when the severity of depressive symptoms is controlled for, approximately 20% of people with nonmalignant chronic pain present with cognitive impairment relative to normative cut-offs [95]. Conversely, a meta-analysis revealed no attention bias towards pain-related information in patients with chronic pain other than CRPS [194].

Although an exhaustive review of neuropsychological changes in chronic pain is beyond the scope of the current article, it is clear that many of the neuropsychological changes reported in CRPS are not unique to this condition. Nonetheless, the therapeutic benefit of treating such changes in CRPS suggests that they are important for understanding its pathology. Furthermore, understanding these cognitive symptoms could potentially result in expanding the neurocognitive treatments that are effective in CRPS to other pain populations.

### 4.4. Targeting Neuropsychological Changes for Treatment of CRPS

The supraspinal mechanisms of CRPS are thought to involve functional cortical reorganization. For instance, the severity of pain and other CRPS signs (mechanical hyperalgesia, tactile discrimination impairment, decreased grip strength, and impaired reach to grasp movements) were related to the extent of functional reorganization of primary sensory and motor cortices [85, 86, 136, 137, 139, 178, 195]. Functional reorganization of the cortical representation of the CRPS-affected limb can be reversed in the course of CRPS treatment [85, 196], and such a reversal is associated with improvement of CRPS symptoms. In one study, the patients who initially showed shrinkage of the cortical representation of the affected limb (relative to unaffected limb and representations of healthy controls) [139] were followed up at least a year later, after successful drug therapy accompanied by physical therapy. Reorganization of the primary somatosensory representations of their CRPS-affected hands was reversed, and this correlated with the extent of the improvement in their CRPS symptoms [196]. Reversal of cortical reorganization of primary and secondary sensory maps was also associated with pain reduction and improved tactile discrimination following drug therapy accompanied by graded desensitisation and motor tasks (sensory-motor returning treatment) [85]. The extent of rereorganization associated with the reduction in CRPS pain suggests that pain is related to the extent of neuroplasticity. Although these findings of cortical reorganization and then normalisation following treatment are only correlational, there is some evidence that targeting the cortical reorganization itself might reduce pain and other symptoms of CRPS. Cortical changes have been targeted directly by anodal transcranial direct current stimulation over primary sensory and motor cortex [197, 198] or repetitive TMS over the motor cortex [199–201]. Both of these interventions resulted in promising analgesic effects in chronic pain, including CRPS in preliminary studies, although the abovementioned studies have not tested whether they actually reverse cortical reorganization.

Compared to direct efforts to induce cortical reorganization, the research on behavioural methods addressing neuropsychological deficits in CRPS has been more extensive. Several therapies, such as mirror therapy, graded motor imagery, and prism adaptation, appear to have beneficial effects on both the neuropsychological and clinical symptoms of CRPS. Mirror visual feedback therapy [202] relies on correcting the mismatch between motor commands and sensory feedback. This method reduced pain and other symptoms, and improved motor function of the affected limb, in people with CRPS with [203–205] and without [46, 206] neurological injury. In graded motor imagery, hand laterality recognition training and imagined hand movements are thought to sequentially activate cortical motor networks without requiring real movements and thus reduce movement-related pain that might be associated with mirror therapy [207–209]. This treatment decreased pain and oedema and reduced the speed of limb laterality recognition in CRPS (although one study failed to replicate the effect of pain reduction [56]). Mirror visual feedback and graded motor imagery can also reduce pain and improve motor function in other chronic pain conditions, particularly phantom limb pain [207, 210, 211]. Prism adaptation [212, 213], adapted from rehabilitation of poststroke neglect, is hypothesised to normalise attention bias and/or the sensory-motor integration system in CRPS. In small uncontrolled studies, it has been shown to reduce subjective body midline bias, body representation distortions, and pain and improve autonomic symptoms and motor function in CRPS [55, 69, 214] (see [215] for a protocol for a randomised controlled trial). Neurorehabilitation has certain advantages over analgesic medications and brain stimulation. For example, it is easily accessible and inexpensive, is not associated with severe side effects, and can be self-administered. However, the neurorehabilitation techniques discussed above are not alternatives to other rehabilitation methods. Instead, they could be used as adjunct therapies to drug treatment, physical/functional therapy, and brain stimulation. Reducing clinical signs such as pain and motor impairment and cognitive symptoms such as body representation distortion can help overcome pragmatic barriers in engaging with traditional rehabilitation.

### 4.5. Summary of Clinical Relevance of Neuropsychological Changes

To summarise, supraspinal mechanisms appear to contribute to CRPS symptomatology on the level of cognitive functions. This is demonstrated by spatial and multisensory modulation of sensory, motor, and autonomic function, and evidence that the extent of neuropsychological changes is related to pain severity. There is emerging support for targeting neuropsychological deficits to relieve physical symptoms of CRPS. Neuroimaging studies indicate that cortical reorganization in CRPS can be reversed, although, thus far, no study has investigated if this reversal is accompanied by any cognitive changes. Conversely, it remains unclear whether neurocognitive treatments reduce the clinical symptoms of CRPS through reversing cortical reorganization or through changes on a behavioural level (or both). In particular, there is currently no neuroimaging research on whether any functional reorganization in parietal networks (implied by neuropsychological changes) relates to clinical manifestations of CRPS. Despite the promising effects of emerging neurorehabilitation strategies, their working mechanisms are yet to be fully understood and the quality of evidence supporting their implementation in standard clinical practice is still insufficient. One potential avenue towards developing new treatments could involve taking advantage of intact cognitive functions. For example, the rubber hand illusion [41] could be used to work towards tolerating touch on the affected limb while observing touch on the artificial limb and altered auditory feedback [48] could be used during auditory-motor adaptation to improve movement of the affected limb.

## 5. Conclusions and Outstanding Questions

Overwhelming evidence of neuropsychological alterations warrants their consideration in the management of CRPS along with the sensory, motor, and autonomic symptoms. Although posttraumatic aberrant inflammatory response can explain several symptoms of CRPS, changes in the central nervous system might better account for these once the peripheral processes subside. The role of cortical mechanisms in CRPS is evident in the neuropsychological symptoms, modulation of low-level sensory and autonomic symptoms by higher cognitive functions (see Table 4), and functional cortical reorganization. Neuropsychological changes found in CRPS include distorted body representation, deficits in lateralised spatial cognition, and impairment of other non-spatially-lateralised cognitive functions (see Table 2). They appear to pertain to manifestation of this syndrome and relate to its clinical outcomes, such as pain. Here, we provide several concluding remarks and lay out suggestions for further research to investigate the cognitive aspects of CRPS and other chronic pain syndromes:

1. The “neglect-like” framework does not fully capture the neuropsychological changes found in CRPS. Instead, disruption to the parietal cortical network might provide a better framework for characterising these symptoms. This would incorporate “neglect-like” symptoms that are often reported in CRPS (which in hemispatial neglect are often associated with temporoparietal right hemisphere lesions [109–111]). However, the parietal framework would also include other changes in spatial cognition that are not consistent with reduced attention to the affected relative to unaffected side (e.g., the shift of the egocentric reference frame towards the affected side [68, 70] or a leftward spatial bias regardless of which side is affected by CRPS [40, 66]). The posterior parietal cortex has been implicated as a crucial area for constructing spatial representations of the body and external space, as well as body ownership [104, 216–219]. Other cognitive changes reminiscent of parietal deficits that have been seen in people with CRPS include impaired non-spatially-lateralised constructional and gnostic abilities [65, 71, 220], although some parietal functions such as multisensory integration might be intact [41, 104]. Overall, combined evidence of abnormal lateralised spatial cognition, body representation, and non-spatially-lateralised cognitive functions in CRPS suggests that functional reorganization of the parietal cortex could underlie the manifestation of neuropsychological symptoms in CRPS. Further neuroimaging studies could test whether functional alterations in parietal cortex indeed correlate with observed neuropsychological symptoms to complement the behavioural findings
2. Neuropsychological symptoms might not all be specific to CRPS, but instead could have ramifications for understanding the cognitive aspects of other chronic pain conditions and applying neurocognitive treatments that are beneficial for CRPS to these disorders. Chronic pain in general can impair cognitive functions such as memory, attention, or executive function, and these impairments have been linked to pain intensity [95, 193]. There are some cognitive changes that distinguish CRPS from other unilateral limb pain syndromes (such as arthritis or neuropathic pain [35, 70, 186]). Nonetheless, some neuropsychological symptoms are seen across these different pain disorders as well as in people with nonlateralised and widespread pain (such as chronic back pain or fibromyalgia) [190]. There are groups of chronic pain syndromes that are associated with plastic changes in the central nervous system, including phantom limb pain, fibromyalgia, and CRPS [221]. People with these conditions can present with similar distortions of body representation and spatial cognition (e.g., [62, 145, 190, 222, 223]), which inspired therapeutic approaches targeting these symptoms to reduce pain [224]
3. Striking findings that cortical reorganization in CRPS can be reversed after recovery [85, 196] suggest that the central mechanisms of chronic pain can be targeted for treatment. Recognising similarities between mechanisms and symptomatology of different pain syndromes can facilitate broader applications of treatments that are beneficial in some disorders. Several neurocognitive rehabilitation strategies developed for CRPS, or adapted from other neurological or pain conditions, have provided some relief from pain and other symptoms [69, 206, 208]. However, there is a need for studies involving larger patient groups and more rigorous controls to better evaluate the benefits of many of these treatments. Another issue is that studies of treatments that target neuropsychological symptoms or cortical networks rarely evaluate the changes in these factors. Identifying the mechanisms of action of neurocognitive treatments and understanding which neuropsychological symptoms should be targeted for rehabilitation would help to maximise its therapeutic effects. For instance, not all individuals with CRPS present with the same neuropsychological changes, thus stratified management might be most efficient
4. Recognising the limitations of the research reviewed in this article and gaps in our understanding of the neuropsychological aspects of CRPS, we would like to put forward some recommendations that could improve further studies on this topic. Even though there is a body of evidence suggesting systematic neuropsychological changes in CRPS that are apparent on a group level, it would be an overstatement to suggest that all people with CRPS present with such symptoms. High variability in the clinical presentation of CRPS [15] also applies to neuropsychological changes, which do not always replicate across different studies. Some studies (including single cases) might have specifically targeted patients with pronounced impairments (e.g., [53, 54, 65, 71]) or have a high proportion of such patients through a combination of random chance and small sample size. This could lead to overestimating certain neuropsychological symptoms in CRPS. Fortunately, there is an increasing tendency to publish null findings, which should allow a more balanced appraisal of the emerging evidence. Although sample sizes in CRPS research are often limited by the availability of people with this rare condition, large-sample, unbiased studies are needed to establish the prevalence of certain neuropsychological changes and potentially identify the characteristics of subgroups of patients in whom these symptoms are more prominent. This could be achieved by combining research efforts across multiple sites and countries. Longitudinal research tracking cognitive changes throughout the course of CRPS and its recovery could enhance the understanding of how they can contribute to the development and maintenance of the disorder and how stable they are over time. Future research could focus on whether there are any cognitive changes in paediatric CRPS and how they correspond to those found in adults. Neuropsychological symptoms in CRPS typically do not arise from any brain injury (in contrast to, for example, hemispatial neglect); thus, they might be more subtle compared to those seen in neurological disorders. To detect and precisely quantify these symptoms in CRPS, researchers should use sensitive measures (e.g., TOJs). In contrast to some neurological conditions, people with CRPS often have insight into their cognitive problems, especially in body representation. Therefore, self-report measures appear to be useful in capturing these symptoms [35, 43]. However, inconsistencies between self-reported disturbances and the same symptoms measured experimentally suggest that we might lack appropriate methods to quantify these changes in a reliable and objective manner. Some studies fail to verify whether observed neuropsychological symptoms are indeed abnormal (see Table 2). Directly comparing the performance of participants with CRPS and matched healthy controls on the same tests allows appropriate quantification of any deviation from what would be considered a normal performance. This is particularly relevant to studying lateralised spatial attention, as a mild leftward bias (“pseudoneglect” [122]) is often found in neurologically healthy participants. Furthermore, routinely including pain control groups would provide insights into which neuropsychological symptoms are unique to CRPS and which are present in other pain conditions as well. This in turn might facilitate our understanding of any central mechanisms specific to CRPS and the development of more targeted treatments

In summary, CRPS appears to be associated with complex neuropsychological changes that include distortions in body representation, deficits in lateralised spatial cognition, and non-spatially-lateralised higher cognitive functions. Some of these cognitive changes are reminiscent of other neuropsychological syndromes that can follow brain lesions, and some might be associated with chronic pain. We argue that the hemispatial neglect framework is not sufficient to characterise the higher cognitive functions affected in people with CRPS. Emerging findings suggest that the disruption of parietal cortical networks can play a role in the manifestation of these neuropsychological symptoms. Importantly, cognitive changes in CRPS (and potentially other chronic pain conditions) can be targeted for treatment. Further research taken beyond the analogy to hemispatial neglect could provide a better understanding of the neuropsychological components of CRPS and elucidate how cortical changes contribute to clinical symptoms of this debilitating condition.

## Figures and Tables

**(a):** 

(i) Continuous pain disproportionate to any inciting event
(ii) Reporting at least one symptom in at least three (clinical diagnostic criteria) or four (research diagnostic criteria) categories
(iii) Displaying at least one sign at the time of assessment in at least two categories
(iv) Lacking other diagnosis that could better explain the symptoms and signs

**(b):** 

Category	Symptoms/signs
Sensory	(i) Hyperesthesia/hyperalgesia (ii) Allodynia
Vasomotor	(i) Temperature asymmetry (ii) Skin colour changes/asymmetry
Sudomotor/oedema	(i) Sweating changes/asymmetry (ii) Oedema
Motor/trophic	(i) Decreased range of motion (ii) Motor dysfunction (weakness, tremor, and dystonia) (iii) Trophic changes (hair, nails, and skin)

**Table 2:** Summary of neuropsychological functions investigated in people with CRPS in research studies published between July 1995 and June 2019.

Neuropsychological function/symptom	Measure/task	Performance of participants with CRPS^a,b^	Study details^c^
Body representation			

Self-reported body perception	Interview	Distorted representation of the affected limb (altered perceptions of size, shape, and weight; desire to amputate; mismatch between sensations and appearance of the limb; erasure of its anatomical parts; poor awareness of its position; and asomatognosia)	Galer et al. [33], *N* = 11; Lewis et al. [34], *N* = 27
	Neglect-like symptoms questionnaire [35, 36]	Asomatognosia (feelings of foreignness and lack of ownership of the affected limb) (17-90%)	Förderreuther et al. [37], *N* = 40; Frettlöh et al. [35], *N* = 123, PC; Galer and Jensen [36], *N* = 224; Kolb et al. [38], *N* = 20, HC, PC†; Michal et al. [39], *N* = 50, PC; Reinersmann et al. [40], *N* = 24, PC†, [41], *N* = 24, PC; Wittayer et al. [42], *N* = 53
	Bath CRPS body perception disturbance scale [43]	Distorted representation of the affected limb (see above)	Brun et al. [44], *N* = 13; Bultitude et al. [45], *N* = 24; Kotiuk et al. [46], *N* = 50; Lewis and Schweinhardt [47], *N* = 22, HC; Tajadura-Jiménez et al. [48], *N* = 12
Objective limb size	Estimation of actual limb size based on enlarged or shrunk images	Overestimation of size of the affected limb	Moseley [49], *N* = 50, PC, AL; Peltz et al. [50], *N* = 30, HC, AL
	Tactile distance judgements following tool use	Perceived lengthening of the unaffected arm and shortening of the affected arm	Vittersø et al. [51], *N* = 36, HC, BL
Limb position sense	Limb position matching	Reduced accuracy in both limbs	Brun et al. [44], *N* = 13, HC, BL; Lewis et al. [52], *N* = 20, HC, BL
	Manual straight-ahead pointing (eyes closed)	Bias towards the affected side of space	Christophe et al. [53], *N* = 1, NC, BL; Jacquin-Courtois et al. [54], *N* = 1, NC, HC, AL
		Normal	Christophe et al. [55], *N* = 7, NC, BL; Kolb et al. [38], *N* = 20, HC, PC, BL
Limb movement sense	Estimation of the extent of actual movement relative to altered visual feedback	Reduced accuracy and precision in the affected limb	Brun et al. [44], *N* = 13, HC, AL
Mental limb rotation/internal representation of limbs	Limb laterality recognition test	Reduced accuracy for the affected vs. unaffected limb images	Johnson et al. [56], *N* = 29
		Longer reaction times for the affected vs. unaffected limb images	Johnson et al. [56], *N* = 29; Moseley [57], *N* = 18, HC; Reid et al. [58], *N* = 130; Schwoebel et al. [59], *N* = 13, HC, [60], *N* = 12
		Longer reaction times for images of both limbs in the affected vs. unaffected side of space	Reid et al. [58], *N* = 130
		Longer reaction times for images of both limbs	Bultitude et al. [45], *N* = 24, HC; Kohler et al. [61], *N* = 15, HC; Reinersmann et al. [62], *N* = 12, HC, PC†; Wittayer et al. [42], *N* = 53, HC
		Normal	Breimhorst et al. [63], *N* = 20, HC; Reinersmann et al. [40], *N* = 24, HC, PC
Multisensory integration/body ownership	Rubber hand illusion	Normal	Reinersmann et al. [41], *N* = 24, HC, PC, BL
Bimanual representation of limbs	Artificial finger illusion	Reduced illusion strength for vision-proprioception only (abnormal bimanual representation); normal with tactile input	Wang et al. [64]; *N* = 20, HC, BL

Lateralised spatial cognition			

Self-reported motor neglect	Interview/clinical observation	Motor neglect for the affected limb (slower initiation, execution, and decreased amplitude and spatial extent of movements, required directed attention to move the affected limb, and occurrence of involuntary movements)	Galer et al. [33], *N* = 11
	Neglect-like symptoms questionnaire [35, 36]	Motor neglect for the affected limb (see above) (17-90%)	Frettlöh et al. [35], *N* = 123, PC; Galer and Jensen [36], *N* = 224; Kolb et al. [38], *N* = 20, HC, PC†; Michal et al. [39], *N* = 50, PC; Reinersmann et al. [40], *N* = 24, PC†, [41], *N* = 42, PC; Wittayer et al. [42], *N* = 53
Visuomotor spatial attention	Line bisection	Bias towards the affected relative to unaffected side of space	Christophe et al. [53], *N* = 1, NC, BL; Jacquin-Courtois et al. [54], *N* = 1, HC, NC, AL; Förderreuther et al. [37], *N* = 29, HC, BL
		Bias away from the affected relative to unaffected side of space	Robinson et al. [65], *N* = 1, NC
		Normal	Christophe et al. [55], *N* = 7, NC, BL; Förderreuther et al. [37], *N* = 29, HC, BL; Kolb et al. [38], *N* = 20, HC, PC; Reid et al. [58], *N* = 13, NC, BL; Reinersmann et al. [40], *N* = 24, HC, PC
	Robot-assisted line bisection	Bias towards the left relative to right side of space	Verfaille et al. [66], *N* = 15, HC, UL
	Line bisection on the limbs	Bias away from the affected relative to unaffected side of space (on the affected limb and on both limbs on the affected side of space)	Reid et al. [58], *N* = 13, NC, BL
	Clock drawing test	Normal	Kolb et al. [38], *N* = 20, HC, PC
Egocentric frame of reference	Visual subjective body midline	Bias towards the affected relative to unaffected side of space (only in the dark)	Christophe et al. [53], *N* = 1, NC; Jacquin-Courtois et al. [54], *N* = 1, HC, NC; Sumitani et al. [67], *N* = 27, HC [68], *N* = 36, HC, [69], *N* = 5, NC; Uematsu et al. [70], *N* = 22, PC
		Bias towards the left relative to right side of space (in the dark)	Reinersmann et al. [40], *N* = 24, HC, PC
		Normal (in the dark)	Christophe et al. [55], *N* = 7, NC; Wittayer et al. [42], *N* = 53, HC
Tactile spatial attention	Confrontation test (detection of concurrent stimulation on both limbs)	Omissions of stimuli on the affected side of the body (extinction; 14%)	Cohen et al. [71], *N* = 22, BL
	Temporal order judgements	Bias away from the affected relative to unaffected limb (when tactile stimuli delivered to uncrossed hands)	Reid et al. [58], *N* = 13, NC
	Temporal order judgements	Bias away from the affected limb (when tactile stimuli delivered to uncrossed hands) and from the affected side of space (when tactile stimuli delivered to hands crossed over body midline), relative to the unaffected limb and side of space	Moseley et al. [72], *N* = 10, [73], *N* = 10, HC
		Normal (crossed and uncrossed hands)	Filbrich et al. [74], *N* = 12, NC
Auditory spatial attention	Temporal order judgements	Normal	Reid et al. [58], *N* = 13, NC
Visual spatial attention	Temporal order judgements	Bias away from the affected relative to unaffected side of space and limb (when visual stimuli presented in near space without hands, or on the surface of uncrossed hands, but not when hands were crossed over body midline)	Bultitude et al. [45], *N* = 24, HC
		Bias away from the affected relative to unaffected side of space (when visual stimuli presented near uncrossed hands but not far from the hands)	Filbrich et al. [74], *N* = 14, NC
	Orienting saccades to cued and noncued stimuli in the left and right visual fields	Normal	Filippopulos et al. [75], *N* = 8, HC
	Speeded detection task	Longer reaction times in the right side of space	Kolb et al. [38], *N* = 20, HC, PC
Internal representation of space	Mental number line bisection	Deviation away from the affected relative to unaffected side of space	Sumitani et al. [67], *N* = 27, HC
		Deviation towards the affected relative to unaffected side of space	Christophe et al. [53], *N* = 1, NC; Jacquin-Courtois et al. [54], *N* = 1, NC, HC
Spatially-defined motor control	Rhythmic finger tapping	Normal/no hands asymmetry (with one and both hands, in uncrossed and crossed posture, with and without visual feedback)	Christophe et al. [55], *N* = 7, HC, BL
		Normal/no hands asymmetry (with one and both hands, hands close together or further apart, without visual feedback)	Christophe et al. [53], *N* = 1, BL
	Speeded button pressing	Slower and more variable movements (with the affected vs. unaffected hand in both sides of space, and with both hands in the affected vs. unaffected side of space)	Reid et al. [76], *N* = 13, BL
	Circle drawing task	Reduced accuracy (with the affected vs. unaffected hand in both sides of space, and with both hands in the affected vs. unaffected side of space)	Reid et al. [76], *N* = 13, BL
		Normal/no hands asymmetry	Christophe et al. [55], *N* = 7, HC, BL

Non-spatially-lateralised cognition			

Object recognition	Tactile recognition of objects	Astereognosia for the affected hand (64%)	Cohen et al. [71], N=22, HC, BL
	Visual recognition of objects	Normal	Robinson et al. [65], *N* = 1, NC
Face recognition	Benton test of face perception	Prosopagnosia	Robinson et al. [65], *N* = 1, NC
Finger identification	Identification of indicated fingers (verbally, by touch, pointing, or movement)	Finger agnosia on the affected limb (48-59%); longer reaction times, reduced accuracy, and increased variability of finger discrimination (on both hands, but worse on the affected hand)	Cohen et al. [71], *N* = 22, HC, BL; Förderreuther et al. [37], *N* = 73, BL; Kuttikat et al. [77], *N* = 13, HC, BL
		Normal	Robinson et al. [65], *N* = 1, NC, UL
Tactile recognition of writing on the skin	Identification of letters and numbers traced onto one's palm	Dysgraphaesthesia on the affected hand (36%)	Cohen et al. [71], *N* = 22, HC, BL
Constructional ability	Copying or constructing named geometric figures using drawing or matchsticks	Constructional apraxia for the affected hand (32%)	Cohen et al. [71], *N* = 22, HC, BL
	Kohs block test	Normal	Kolb et al. [38], *N* = 20, HC, PC
Numerical and language processing	Counting, mental arithmetic, reading, repeating, writing, copying, identifying numbers and letters/words, spelling	Dyscalculia (27%); dysgraphia for the affected hand (27%)	Cohen et al. [71], *N* = 22, HC, BL
Speech repetition	Repetition of words and sentences, confrontation naming	Conductional dysphasia (4%)	Cohen et al. [71], *N* = 22, HC
Verbal fluency	Boston Naming test, animal (semantic) fluency, letter fluency	Impaired verbal fluency	Libon et al. [78], *N* = 137, NC
Visuospatial orientation	Rod Orientation test	Normal	Kolb et al. [38], *N* = 20, HC, PC
Knowledge about object orientation	Object orientation judgements, copying, drawing, and reorienting objects into upright position	Agnosia for object orientation	Robinson et al. [65], *N* = 1, NC
Knowledge about order and orientation of numbers and letters/words	Spontaneous and dictated writing and copying	Mirror reversal in writing and reading, horizontal inversion of letters and words, and letters and numbers ordering in writing (cases for the affected hand, both hands, and unaffected hand)	Cohen et al. [71], *N* = 22, HC, BL; Robinson et al. [65], *N* = 1, UL
	Letter orientation recognition	Normal (for standard vs. reflected letters and left vs. right side of space)	Reid et al. [58], *N* = 13
Body sides differentiation	Identification of indicated body parts (verbally, by touch, or pointing)	Left-right disorientation (9%)	Cohen et al. [71], *N* = 22, HC, BL
		Normal	Robinson et al. [65], *N* = 1, NC, UL
Imitation of complex movements	Pantomime of indicated motor acts	Ideomotor apraxia (5%)	Cohen et al. [71], *N* = 22, HC, BL
Temporal acuity	Temporal order judgements	Reduced temporal acuity	Bultitude et al. [45], *N* = 24, HC
Alertness	Test of attentional performance	Normal response readiness	Reinersmann et al. [62], *N* = 12; HC, PC
Working memory	Digit span	Impaired working memory span	Libon et al. [78], *N* = 137, NC
	Test of attentional performance	Normal continuous updating	Reinersmann et al. [62], *N* = 12, HC, PC
Spatial working memory	Block tapping test	Normal	Kolb et al. [38], *N* = 20, HC, PC, right limb
Episodic verbal memory and learning	California verbal learning test II	Impaired encoding, recall, and recognition	Libon et al. [78], *N* = 137, NC
Global cognitive processing	Digit span, Boston naming test, animal (semantic) fluency, letter fluency, and California verbal learning test II	Global processing impairment (particularly impaired naming, declarative memory, and executive function; 23%) or mild dysexecutive syndrome (particularly impaired working memory and verbal fluency; 42%)	Libon et al. [78], *N* = 137, NC

^a^Percentages represent the proportion of individuals with CRPS out of the total CRPS sample who presented with abnormal performance. We reported percentages where available; in other cases, we presented group effects. ^b^Normal performance indicates that there were no differences between participants with CRPS and control participants and/or between the affected and unaffected side among participants with CRPS. ^c^*N* represents CRPS sample size. Where applicable, we specified which control group was included (HC = healthy/pain-free controls; PC = pain controls; NC = normative data or comparison against zero; † = no significant difference between CRPS and control group) and which limb(s) were tested (AL = affected limb; UL = unaffected limb; BL = both limbs).

**Table 3:** Poststroke hemispatial neglect symptoms.

Domains	Categories	Deficits
Modality	Perceptual neglect	Difficulty with allocating attention to visual, tactile, or auditory stimuli appearing on the contralesional side of space
	Motor neglect	Reduced or slowed movements using the contralesional limb that cannot be attributed to primary motor deficit; reduced or slowed movements in/towards the contralesional side of space
	Representational neglect	Problems imagining or visualising the contralesional side of scenes

Reference frame	Egocentric	Underrepresentation of contralesional side of space in relation to one's own body/body parts (e.g., subjective estimate of one's body midline or straight ahead shifted towards the ipsilesional side)
	Allocentric	Underrepresentation of contralesional side of spatial relationships between external objects separated in space (e.g., bisections of straight line shifted toward the end corresponding to the ipsilesional side)

Region of space	Personal	Reduced attention to contralesional side of the body
	Peripersonal	Reduced attention to contralesional side of the space within one's reach
	Extrapersonal	Reduced attention to contralesional side of the space beyond one's reach

**Table 4:** Evidence of modulation of low-level sensory and autonomic functions in CRPS by spatial or multisensory manipulations.

Function	Manipulation	Affected low-level sensory/autonomic/motor function in people with CRPS^a^	Study details^b^
Visual perception	Viewing ambiguous/conflicting visual stimuli	Increased pain (61-73%), sensory disturbances (73%), dystonia (33%) in the affected limb, and asymmetric vasomotor response (34%)	Cohen et al. [184], *N* = 30, HC, BL; Hall et al. [185], *N* = 30, HC, PC
Auditory perception	Hearing uncomfortably loud sound	Painful sensations to sound (hyperacusis; 38%)	de Klaver et al. [187], *N* = 40
Sensory-motor integration	Incongruent mirror visual feedback during active movements	Increased pain and sensory disturbances	Brun et al. [186], *N* = 38, HC, PC, BL
Tactile perception	Mirror visual feedback of stimulated unaffected limb	Pain and paraesthesia experienced in the corresponding location on the nonstimulated affected limb (allochiria); cold perceived concurrently on the stimulated and nonstimulated limb (dysynchiria)	Acerra and Moseley [188], *N* = 10, HC, PC, UL
Temperature modulation	Physically resting or viewing the affected limb as positioned in the unaffected side of space through prism glasses	Normalization of temperature asymmetry between the limbs	Moseley et al. [182], *N* = 10, HC, BL, [72], *N* = 23, HC, BL
Visual perception	Viewing enlarged image of the affected limb through magnifying lenses or in virtual environment or shrunk images of affected limb through minifying lenses	Pain and swelling (evoked by movement) increased when viewing enlarged image, reduced when viewing shrunken image	Matamala-Gomez et al. [189], *N* = 9, PC, AL; Moseley et al. [183], *N* = 10, AL

^a^Percentages represent the proportion of individuals with CRPS out of the total CRPS sample who presented with abnormal performance. We reported percentages where available; in other cases, we presented group effects. ^b^*N* represents CRPS sample size. Where applicable, we specified what control group was included (HC = healthy/pain-free controls; PC = pain controls) and which limb(s) were tested (AL = affected limb; BL = both limbs).
